# The Free Radical Scavenging and Anti-Isolated Human LDL Oxidation Activities of *Pluchea indica* (L.) Less. Tea Compared to Green Tea (*Camellia sinensis*)

**DOI:** 10.1155/2020/4183643

**Published:** 2020-09-25

**Authors:** Kittipot Sirichaiwetchakoon, Gordon Matthew Lowe, Griangsak Eumkeb

**Affiliations:** ^1^School of Preclinic, Institute of Science, Suranaree University of Technology, 111 University Avenue, Suranaree Subdistrict, Muang District, Nakhonratchasima 30000, Thailand; ^2^School of Pharmacy and Biomolecular Sciences, Liverpool John Moores University, James Parsons Building, Byrom Street, Liverpool, UK

## Abstract

Tea is one of the most popular beverages in the world. *Camellia sinensis* tea (CST) or green tea is widely regarded as a potent antioxidant. In Thailand, *Pluchea indica* (L.) Less. tea (PIT) has been commercially available as a health-promoting drink. This study focused on free radical scavenging activities of PIT, and its ability to protect isolated human low-density lipoproteins (LDL) from oxidation by chemical agents. A preliminary study to investigate the antioxidant nature of PIT was undertaken. These included common antioxidant assays involving 2,2-Diphenyl-1-picrylhydrazyl (DPPH), 2,2-azinobis-(3-ethylbenzothiazoline)-6-sulfonic acid (ABTS), hypochlorous acid (HOCl), and its potential to scavenge peroxynitrite. In separated experiments, isolated human LDL was challenged with either 2,2′-azobis(2-amidinopropane) dihydrochloride (AAPH), copper (Cu^2+^), or 3-Morpholinosydnonimine hydrochloride (SIN-1) to induce LDL oxidation. PIT exhibited antioxidant activity in all test systems and performed significantly better than CST in both DPPH (*P* < 0.05; IC_50_PIT = 245.85 ± 15.83 and CST = 315.41 ± 24.18 *μ*g/ml) and peroxynitrite scavenging assays. PIT at 75 *μ*g/ml almost fully prevented the peroxynitrite over a 5 h period. Moreover, it displayed similar properties to CST during the antioxidation of isolated human LDL using AAPH, Cu^2+^, SIN-1, and hypochlorous acid scavenging assays. However, it revealed a significantly lower ABTS scavenging activity than CST (*P* < 0.05; IC_50_PIT = 30.47 ± 2.20 and CST = 21.59 ± 0.67 *μ*g/ml). The main constituents of the PIT were identified using LC-MS/MS. It contained 4-O-caffeoylquinic acid (4-CQ), 5-O-caffeoylquinic acid (5-CQ), 3,4-O-dicaffeoylquinic acid (3,4-CQ), 3,5-O-dicaffeoylquinic acid (3,5-CQ), and 4,5-O-dicaffeoylquinic acid (4,5-CQ). In conclusion, caffeoyl derivatives in PIT could play an important role in potent antioxidant properties. So, it may be further developed to be antioxidant beverages for preventing atherosclerosis and cardiovascular diseases associated with oxidative stress.

## 1. Introduction

Oxidative stress is defined as the imbalance between the production of free radicals and defense mechanisms, which are natural physiological processes in biological systems [1]. The excesses of intracellular reactive oxygen species (ROS) and reactive nitrogen species (RNS) is the major causes of oxidative stress that is associated with the development of chronic and degenerative diseases such as cancer, arthritis, aging, autoimmune disorders, cardiovascular, and neurodegenerative diseases [2–5]. Moreover, oxidative stress may also modify the structure and function of certain biomolecules, including proteins, lipids, and DNA [6].

Oxidative stress may also result in the oxidation of human LDL. LDL oxidation may result in lipid peroxidation or the direct oxidation of apolipoprotein. Oxidized LDL is thought to have a vital role in the etiology of atherosclerosis, which ultimately has a profound effect on cardiovascular function [7–9].

Herbal supplements derived from fruit and vegetables tend to be rich in both water and lipid-soluble antioxidants [10]. One of the most popular herbal supplements is a beverage, such as a tea infusion. Many herbal tea infusions have a strong antioxidant capacity [11–14], and it has the potential to prevent diseases associated with oxidative stress such as atherosclerosis [15, 16].

Tea is the most widely consumed beverage in the world, second only to water [17], and one of the most popular beverages in Southeast Asia. Green tea is derived from the tea plant *Camellia sinensis*. It has been demonstrated that some of the components of green tea have potent antioxidative properties and have free radical scavenging properties towards the DPPH, ABTS, and Fluorescence Recovery After Photobleaching (FRAP) assays [18]. Also, flavonols from green tea have potent antioxidant capacities and reduced oxidative stress [19]. Moreover, green tea can prevent lipid oxidation induced by copper ions [20]. The main active ingredients of green tea are polyphenol catechins. The major catechins present in green tea are Epicatechin (EC), Epigallocatechin gallate (EGCG), Epicatechin gallate (ECG), Epigallocatechin (EGC), and Gallocatechin gallate (GCG) [21]. EGCG, which acts as an antioxidant, might exert a preventive effect against cardiovascular disease [22].

The plant *Pluchea indica* (L.) Less. (*P. indica*) is a large evergreen shrub found abundantly in salt marshes. It is widely distributed in India, Southern China, and Southeast Asia. In Thailand, The PIT has been commercially available for approximately ten years as a health-promoting drink [23]. The various biological activities of *P. indica* leaves have been widely reported. Several studies revealed that its methanolic extract had diuretic [24], hypoglycemic, and antihyperglycemic effects [25]. Moreover, the ethyl acetate fraction from an ethanolic extract of *P. indica* in lipopolysaccharide- (LPS-) stimulated RAW 264.7 macrophages displayed anti-inflammatory activities [26]. Besides, the volatile oil of *P. indica* exhibited antioxidant activity [27]. Although some biological activities of *P. indica* have been reported, there is little information published on the antioxidative properties of the aqueous tea.

The aim of this study was to examine the free radical scavenging potential of PIT with regard to established antioxidant assays and its potential to inhibit the oxidation of isolated human LDL by Cu^2+^, AAPH, or SIN-1. The antioxidant properties of PIT were compared to a commercially available green tea.

## 2. Materials and Methods

### 2.1. Tea Materials

PIT was supplied by the Crystal Biotechnology Company, Thailand. Commercial green tea was purchased from a supermarket in the United Kingdom. The tea samples were kept in the dark, dry place until required. Beverages were prepared by brewing ground tea leaves in 80°C 1x phosphate buffer saline (PBS) for 5 min and filtered by Whatman No. 1 filter paper. The concentration of PIT was calculated from the ground tea leaves dry weight in PBS volume (*μ*g/ml). Tea samples were kept at -20°C until used.

### 2.2. Chemicals and Reagents

2,2-Diphenyl-1-picrylhydrazyl (DPPH), 2,2-azinobis-(3-ethylbenzothiazoline)-6-sulfonic acid (ABTS), potassium persulfate, sodium hypochlorite (NaOCl), catalase, sulfanilamide, naphthylethylenediamine dihydrochloride (NED), 3-Morpholinosydnonimine hydrochloride (SIN-1), Diethylene-triamine-pentaacetic acid (DTPA), Evans blue, iodixanol (Optiprep™), 2,2′-azobis (2-amidinopropane) dihydrochloride (AAPH), copper sulfate (Cu_2_SO_4_), trichloroacetic acid (TCA), thiobarbituric acid were purchased from Sigma-Aldrich Chemical Co. (St. Louis, MO, USA). 4-O-caffeoylquinic acid (4-CQ), 5-O-caffeoylquinic acid (5-CQ), 3,4-O-dicaffeoylquinic acid (3,4-CQ), 3,5-O-dicaffeoylquinic acid (3,5-CQ), and 4,5-O-dicaffeoylquinic acid (4,5-CQ) were purchased from Chengdu Biopurify Phytochemicals Ltd., China. Other reagents used were all analytical grade.

### 2.3. DPPH Scavenging Assay

The %DPPH scavenging activity of PIT and CST were evaluated by the method of Brand-Williams et al. [28] with slight modifications. In brief, 0-300 *μ*g/ml of the tea extracts was prepared using 0.002% DPPH reagent in a 96-well plate. The samples were suitably mixed prior to incubation in the dark at room temperature for 30 min. PBS was used as a negative control. The absorbance of all samples was measured at 515 nm. Radical scavenging activity was repeated for six times and expressed as a DPPH scavenging percentage as,

(1) $$\begin{array}{c} \%\text{DPPH scavenging}=\left(1-\frac{{\text{OD}}_{\text{sample}}-{\text{OD}}_{\text{sample blank}}}{{\text{OD}}_{\text{control}}-{\text{OD}}_{\text{sample blank}}}\right)\times 100. \end{array}$$

### 2.4. ABTS Scavenging Assay

The ABTS scavenging activity of PIT was measured and compared with CST. The ABTS^+^ radical cation was prepared by mixing 7 mM ABTS stock solution with 2.45 mM potassium persulfate (final concentration) in methanol and kept them in the dark at room temperature for 16 h. The solution was adjusted to an absorbance of 0.70 (±0.02) with ethanol. The reaction was performed in a 96-well plate by mixing and shaking 90 *μ*l ABTS^+^ radical cation solution with 10 *μ*l of the test sample at various concentrations (0–40 *μ*g/ml final concentration) for 45 seconds. The absorbance was measured at 734 nm. The assay was repeated six times, and the percentage inhibition of absorbance was reported as an ABTS scavenging percentage as

(2) $$\begin{array}{c} \%\text{ABTS scavenging}=\left(1-\frac{{\text{OD}}_{\text{sample}}-{\text{OD}}_{\text{sample blank}}}{{\text{OD}}_{\text{control}}-{\text{OD}}_{\text{sample blank}}}\right)\times 100. \end{array}$$

### 2.5. Hypochlorous Acid (HOCl) Scavenging Assay

This assay was performed as previously described by Aruoma and Halliwell [29] with minor modifications. HOCl was generated by adjusting the pH of a 10% (*v*/*v*) solution of NaOCl to 6.2% with 0.6 M H_2_SO_4_. The HOCl was mixed with 50 mM phosphate buffer (pH 6.8), catalase (7.2 *μ*M), and tea samples at various concentrations (0–300 *μ*g/ml) in 96 well-plate. The mixture was incubated at 25°C for 20 min. The scavenging activity was measured by the decrease in absorbance of catalase at 404 nm. The assay was performed in triplicate. %HOCl scavenging was calculated as

(3) $$\begin{array}{c} \%\text{HOCl scavenging}=\left(1-\frac{{\text{OD}}_{\text{sample blank}}-{\text{OD}}_{\text{sample}}}{{\text{OD}}_{\text{sample blank}}-{\text{OD}}_{\text{control}}}\right)\times 100. \end{array}$$

### 2.6. Nitric Oxide (NO) Scavenging Assay

SIN-1 was used for generating RNS in this experiment. One of the end products of RNS chemistry is nitrite. Nitrite concentration was determined using the Griess assay. The test solution was prepared in a 96-well plate by mixing 0.25 mM SIN-1 with PBS (pH 7.4) and various doses of tea samples (0–100 *μ*g/ml final concentration). The test solutions were incubated at 25°C for 30 min. Then, 80 *μ*l of 0.33% sulfanilamide in 20% glacial acetic acid was added to the produced solution and was suitably shaken for 5 min before adding 80 *μ*l of 0.1% NED and incubated at 25°C for 15 min. The nitric oxide scavenging was measured spectrophotometrically at 540 nm against a blank sample. All tests were repeated six times, and %Nitric oxide radical scavenging was expressed as

(4) $$\begin{array}{c} \%\text{Nitric oxide radical scavenging}=\left(1-\frac{{\text{OD}}_{\text{sample}}-{\text{OD}}_{\text{sample blank}}}{{\text{OD}}_{\text{control}}-{\text{OD}}_{\text{sample blank}}}\right)\times 100. \end{array}$$

### 2.7. Peroxynitrite Scavenging Assay

SIN-1 was used as a peroxynitrite donor, and peroxynitrite scavenging activity was measured by an Evans blue bleaching assay. Briefly, the reaction mixture comprised of 50 mM phosphate buffer (pH 7.4), 0.1 mM DTPA, 90 mM NaCl, 5 mM KCl, 12.5 *μ*M Evans blue, 1 mM SIN-1, 37.5, and 75 *μ*g/ml of either PIT or CST in a 96-well plate. The mixture was incubated at 37°C for 300 min, and the absorbance was measured at 611 nm every 30 min. The assay was performed six times. The percentage scavenging of peroxynitrite at various times was calculated by the %Optical density of Evans blue as;

(5) $$\begin{array}{c} \%\text{Optical density of Evans blue}=\left(1-\frac{{\text{OD}}_{\text{sample blank}}-{\text{OD}}_{\text{sample}}}{{\text{OD}}_{\text{sample blank}}-{\text{OD}}_{\text{control}}}\right)\times 100. \end{array}$$

### 2.8. LDL Isolation by Ultracentrifugation Technique

The Liverpool John Moores University (LJMU) ethics committee approved the use of human blood and the preparation of LDL. Whole blood was obtained from the vein of healthy volunteers aged between 24 and 70 years who were normolipidemic, nonsmoking, had not taken any medications or supplements within the last two weeks. Whole blood was added to 3.8% (*w*/*v*) sodium citrate at a ratio of blood against anticoagulant as 9 : 1. The whole blood was centrifuged at 1500 x g for 20 min, and the platelet-poor plasma was transferred to a separate plastic tube. LDL was isolated by density gradient ultracentrifugation using a method developed by Graham et al. (1996) with minor modifications [30]. In brief, 0.5 ml of 60% (*v*/*v*) iodixanol (Optiprep™) was mixed with 4.5 ml of plasma and transferred to an 11.2 ml capacity Optiseal tube. Then, it was overlayered with 5 ml of 12% *v*/*v* iodixanol prepared with human plasma, and PBS was used to fill the rest of the tube. The tubes were centrifuged in a Beckman L8-80 ultracentrifuge using a vertical rotor V65.1 at 350,000 x g for 3 h. An Auto Densi-Flow gradient fractionator (Labconco, UK) was used for fractionating the gradient by unloading the gradient from the top to the bottom of the tube. Each tube of 11.2 ml was fractionated into 0.5 ml aliquots per tube. All fractions were measured for triglycerides, cholesterol, LDL, and apoB100 components by using reagents and standards from Randox (Eire). Pooled LDL fraction was measured protein concentration by Bradford assay and stored at -20°C until required.

### 2.9. AAPH Induce LDL Oxidation Assay

AAPH is a reactive oxygen species generator. The sample solution was prepared by adding 20 mM AAPH to 50 *μ*g/ml of LDL protein. Either 50 *μ*g/ml or 75 *μ*g/ml of PIT or CST was added in the test samples. The control sample was 50 *μ*g/ml of LDL protein added with PBS. The LDL protein was added with AAPH 20 mM. All samples were incubated at 37°C. At this temperature, AAPH produces a range of ROS. At a specific time point (0, 30, 60, 90, 120, 150, and 180 min), sample aliquots were removed and placed at -20°C prior to thiobarbituric acid reactive substances (TBARS) measurements. The first stage of the TBARS assay was to precipitate protein by the addition of 20% *v*/*v* TCA. The samples were then spun at 10,000 rpm for 10 min at 4°C. The supernatant was collected and treated with 1% *w*/*v* thiobarbituric acid. The samples were heated at 95°C for 20 min. TBARS concentration was determined by UV absorption at 532 nm. The malondialdehyde (MDA) concentration was determined by a calibration curve. The assay was performed in triplicate.

### 2.10. Copper Induce LDL Oxidation Assay

LDL protein was challenged with Cu^2+^ by adding Cu_2_SO_4_ at a final concentration of 40 *μ*M to 50 *μ*g/ml of LDL protein. The negative control sample was 50 *μ*g/ml of LDL protein with PBS. The positive control was 50 *μ*g/ml of LDL protein incubated with 40 *μ*M Cu_2_SO_4_. The 15 *μ*g/ml of PIT or CST was added in the test samples that contained 50 *μ*g/ml LDL protein plus 40 *μ*M Cu_2_SO_4_. All samples were incubated at 37°C for 3 h. At various time points, the samples were assessed for MDA equivalence as TBARS products. The assay was performed in triplicate.

### 2.11. SIN-1 Induce LDL Oxidation Assay

SIN-1, which is a peroxynitrite donor, has been used to investigate RNS-mediated LDL oxidation. In this assay, 50 *μ*g/ml of LDL protein was challenged with 1 mM of SIN-1. In test samples, 15 *μ*g/ml of PIT or CST was mixed with 50 *μ*g/ml of LDL protein and 1 mM of SIN-1. The samples were incubated at 37°C for 18 h. Finally, the samples were stored at -20°C before further analysis by TBARS assay. The assay was repeated three times.

### 2.12. LC-MS/MS Instrument and Conditions

LC-MS/MS technique has been used for identifying the main chemical constituents of PIT as previously described by Kongkiatpaiboon et al. [23]. The combination of chromatographic separation of LC-MS/MS system was combined by Agilent HPLC 1290 Infinity and mass analyzer 6490 Triple Quad LC/MS Agilent Technologies, which equipped with an electrospray ionization (ESI) source system. Agilent ZORBAX Rapid Resolution High Definition (RRHD) SB-C18, 2.1 mm id × 150 mm (1.8 *μ*m) was used for chromatographic separation. The mobile phase system used 1% formic acid in water as solvent A and 1% formic acid in acetonitrile as solvent B. The gradient of the mobile phase was set at a ratio of solvent A : solvent B, 100 : 0, with gradient elution, from 30% solvent B at 10 min and 100% solvent B at 30 min at a flow-rate of 0.2 ml/min. The sample injection volume was 5 *μ*L, and the column was set at 25°C. 4-CQ, 5-CQ, 3,4-CQ, 3,5-CQ, and 4,5-CQ were used as standard.

### 2.13. Statistical Analysis

All the data were presented as a mean ± standard deviation (S.D.) or standard error of the mean (S.E.M.). Statistical analysis was performed using SPSS version 18.0. The significant statistical differences between groups of DPPH, ABTS, HOCl, and NO scavenging assay were analyzed by an independent *t*-test, whereas peroxynitrite, Cu^2+^, AAPH, and SIN-1 scavenging assay were compared by one-way analysis of variance (ANOVA) with a Tukey's HSD post hoc test. Values were considered statistically significant when *P* < 0.05, and data were the representative of at least three independent experiments.

## 3. Results

### 3.1. DPPH Scavenging Assay

**Figure 1:**
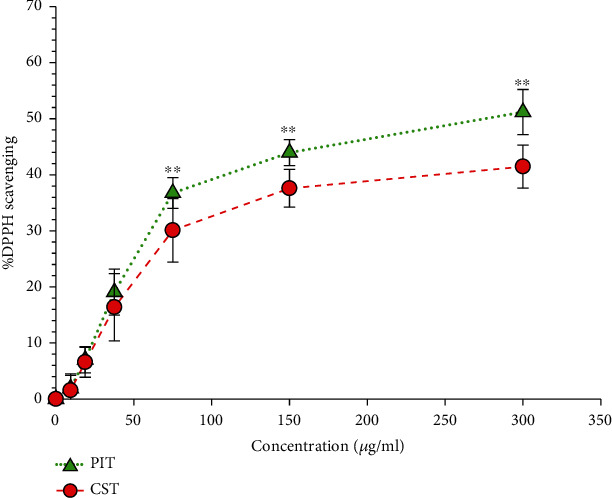
The DPPH radical scavenging activity. PIT: *P. indica* (L.) Less. tea; CST: *C. sinensis* tea at concentrations ranging from 0-300 *μ*g/ml. The data represent the percentage of DPPH inhibition. Each value represents mean ± S.D. (*n* = 6). ^∗∗^ indicates a significant difference between groups at *P* < 0.01.

The activities of both teas were investigated using the DPPH radical scavenging assay. The antioxidant activity was calculated spectrophotometrically at 515 nm. The result indicated that PIT generated significantly stronger antioxidant capacity than CST at a concentration of 75 *μ*g/ml to 300 *μ*g/ml CST (*P* < 0.05) (Figure 1). At a concentration of 300 *μ*g/ml, the %DPPH scavenging value of PIT and CST was 51.19 ± 4.02 and 41.46 ± 3.83, respectively. The IC_50_ value of PIT was determined at 245.85 ± 15.83 *μ*g/ml, which was lower than CST at 315.41 ± 24.18 *μ*g/ml.

### 3.2. ABTS Scavenging Assay

**Figure 2:**
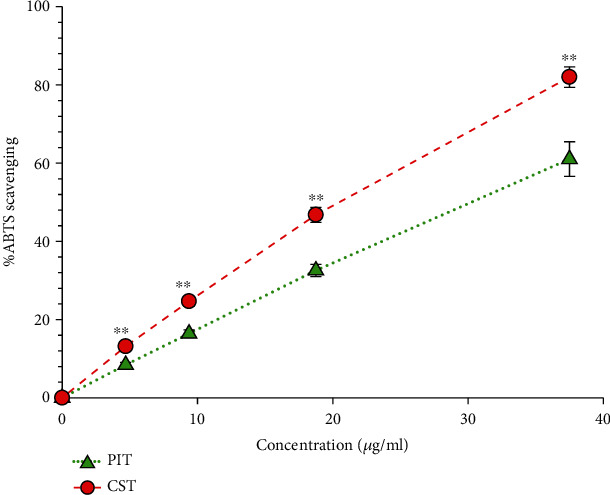
The ABTS radical scavenging activity. PIT: *P. indica* (L.) Less. tea; CST: *C. sinensis* tea at concentrations ranging from 0-40 *μ*g/ml. The data represent the percentage of ABTS inhibition. Each value represents mean ± S.D. (*n* = 6). ^∗∗^ indicates a significant difference between groups at *P* < 0.01.

This assay shows the abilities of the extract to quench the ABTS^+^ radical. The extracts interacted with ABTS^+^, which decreased the absorbance of the solution. The absorbance was measured spectrophotometrically at 734 nm.  Figure 2 displays %ABTS radical scavenging of PIT and CST. CST significantly inhibited ABTS^+^ radical stronger than PIT at 5-40 *μ*g/ml (*P* < 0.05). The IC_50_ of PIT and CST was 30.47 ± 2.20 and 21.59 ± 0.67 *μ*g/ml, respectively.

### 3.3. Hypochlorous Acid Scavenging Assay

**Figure 3:**
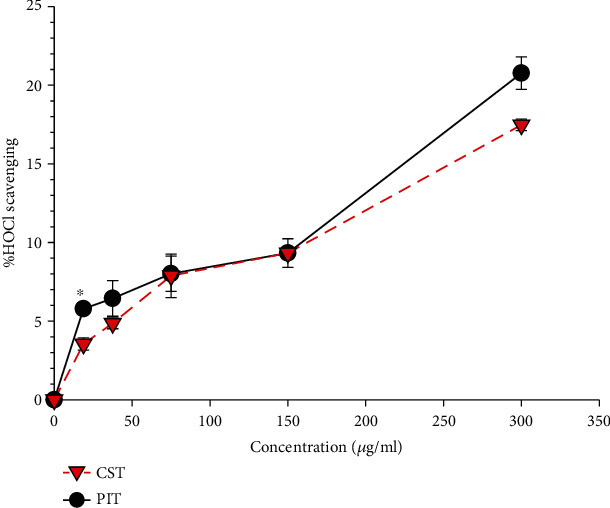
The hypochlorous acid radical scavenging activity. PIT: *P. indica* (L.) Less. tea; CST: *C. sinensis* tea at concentrations ranging from 0-300 *μ*g/ml. The data represent the percentage of HOCl inhibition. Each value represents mean ± S.E.M (*n* = 3). ^∗^ indicates a significant difference between groups at *P* < 0.05.

The hypochlorous acid scavenging activity of the PIT and CST are shown in Figure 3. The results showed that PIT significantly scavenged more hypochlorous acid than CST at a concentration of 18.75 *μ*g/ml (*P* < 0.05). Nevertheless, the other strengths of PIT inhibited more hypochlorous acid than CST but not significantly.

### 3.4. Nitric Oxide Scavenging Assay

**Figure 4:**
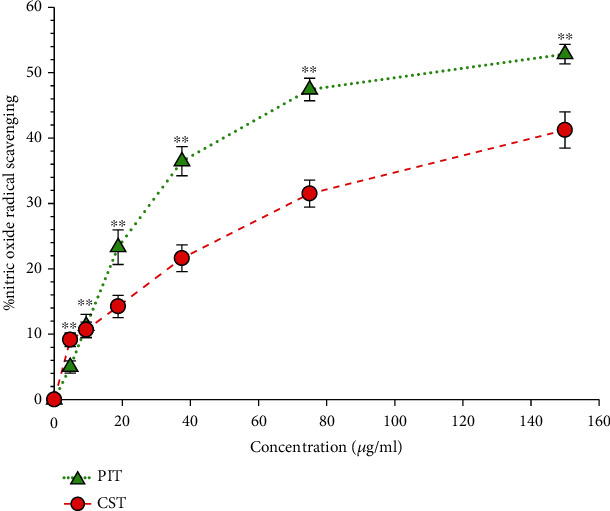
The nitric oxide radical scavenging activity. PIT: *P. indica* (L.) Less. tea; CST: *C. sinensis* tea at concentrations ranging from 0-150 *μ*g/ml in various concentrations. The data represent the percentage of nitric oxide inhibition. Each value represents mean ± S.D. (*n* = 6). ^∗∗^ indicates a significant difference between groups at *P* < 0.01.

Nitric oxide scavenging could be detected by determining the nitrite concentration using the decolorization by the Griess reaction method.  Figure 4 shows % inhibition of nitric oxide of PIT and CST. The result indicated that the %nitric oxide scavenging activity of PIT was significantly higher than CST (*P* < 0.01). The IC_50_ of PIT was 116.48 ± 5.08 *μ*g/ml, while the IC_50_ of CST was 178.42 ± 15.52 *μ*g/ml.

### 3.5. Peroxynitrite Scavenging Assay

**Figure 5:**
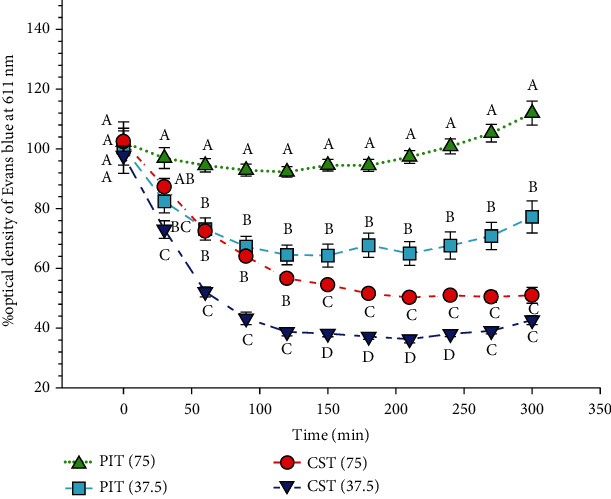
The peroxynitrite radical scavenging activity. PIT(75): *P. indica* (L.) Less. tea at 75 *μ*g/ml; CST(75): *C. sinensis* tea at 75 *μ*g/ml. The data represent the percentage of the optical density of Evans blue at 611 nm at various times over 5 h. Means ± S.E.M is illustrated for six replicates. Means with the same superscript are not significantly different from each other (Tukey's HSD test, *P* < 0.05).

Evans blue assay is used to measure peroxynitrite scavenging, which is generated by SIN-1. The peroxynitrite is thought to bleach the Evans blue dye. The results are shown in Figure 5. At higher concentrations, PIT (75 *μ*g/ml) almost fully prevented the peroxynitrite bleaching the dye over a 5 h period. Whereas at 37.5 *μ*g/ml, the %peroxynitrite scavenging was 64.50 ± 8.07% at 2 h. In contrast, a higher concentration of CST (75 *μ*g/ml) exhibited significantly lower peroxynitrite scavenging activity compared to PIT.

### 3.6. AAPH Induce LDL Oxidation Assay

**Figure 6:**
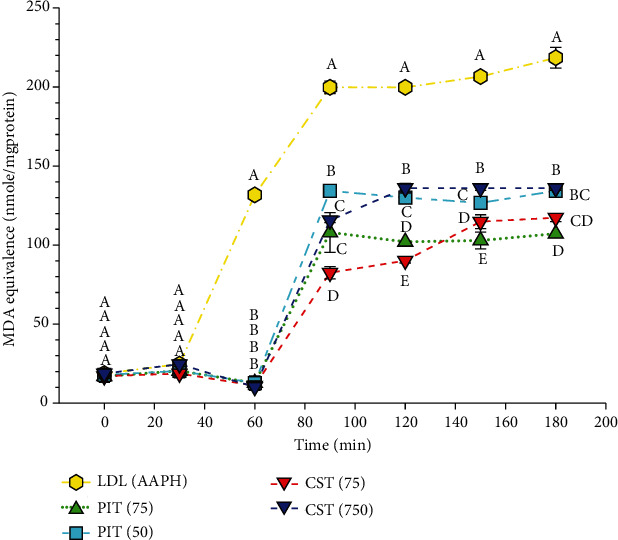
The AAPH induces LDL oxidation scavenging activity. LDL(AAPH): LDL-treated with 20 mM of AAPH; PIT(75): *P. indica* (L.) Less. tea at 75 *μ*g/ml; CST(75): *C. sinensis* tea at 75 *μ*g/ml. The data represent the MDA equivalence at various times over 3 h. Means ± S.D. is illustrated for three replicates. Means with the same superscript are not significantly different from each other (Tukey's HSD test, *P* < 0.05).

The ROS induce LDL oxidation scavenging effect has been investigated by challenging isolated human LDL with AAPH, which is ROS. PIT and CST at 50 and 75 *μ*g/ml were used for the experiment. The samples were incubated for 3 h, and aliquots were collected at various times for analysis using the TBARS assay. The result indicated that after 60 min of AAPH activity, both teas could significantly decrease the TBARS formation (Figure 6). Furthermore, a stronger effect was observed at higher concentrations of both teas. The lag time of LDL oxidation was increased from 40 to 70 min at all strengths of testing teas. These results suggest that both teas have approximately similar LDL oxidation scavenging properties.

### 3.7. Copper Induce LDL Oxidation Assay

**Figure 7:**
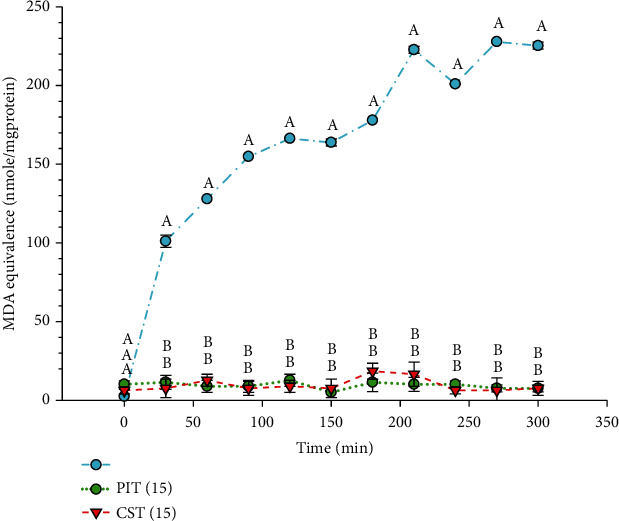
The copper induces LDL oxidation scavenging activity. LDL(Cu^2+^): LDL-treated with 40 *μ*M of copper sulfate; PIT(15): *P. indica* (L.) Less. tea at 15 *μ*g/ml; CST(15): *C. sinensis* tea at 15 *μ*g/ml. The data represent the MDA equivalence at various times over 5 h. Means ± S.D. is illustrated for three replicates. Means with the same superscript are not significantly different from each other (Tukey's HSD test, *P* < 0.05).

Isolated human LDL was incubated with either PIT or CST at 15 *μ*g/ml and challenged with Cu^2+^. The result was presented as MDA equivalence (nmol/mgprotein), which was calculated from the MDA calibration standard curve.  Figure 7 showed that in the Cu^2+^-treated group, the MDA equivalence was continually increased by 210 min at 222.73 ± 2.22 nmol/mgprotein, while the PIT and CST treated group could almost entirely prevent Cu^2+^ oxidation of human LDL.

### 3.8. SIN-1 Induce LDL Oxidation Assay

**Figure 8:**
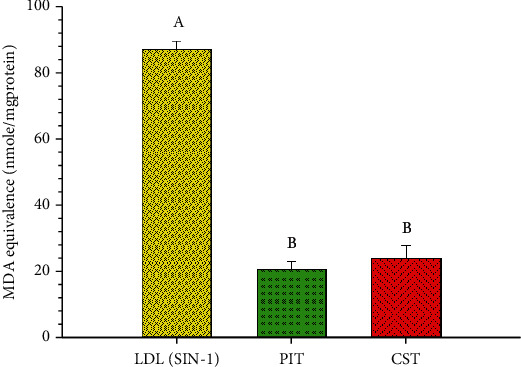
The SIN-1 induces LDL oxidation scavenging activity. LDL(SIN-1): LDL-treated with 1 mM of SIN-1; PIT: *P. indica* (L.) Less. tea at 15 *μ*g/ml; CST: *C. sinensis* tea at 15 *μ*g/ml. The data represent the MDA equivalence at 18 h. Means ± S.D. is illustrated for three replicates. Means with the same superscript are not significantly different from each other (Tukey's HSD test, *P* < 0.05).

SIN-1, which is an RNS generator, is used to induce LDL oxidation. Isolated human LDL was incubated with SIN-1 and tea extracts. The result expressed the RNS scavenging effect of both PIT and CST at a dose of 15 *μ*g/ml (Figure 8). PIT and CST displayed nearly similar properties and could decrease MDA concentration from 86.73 ± 2.55 to 20.41 ± 2.55 and 23.81 ± 3.90 nmol/mgprotein, respectively.

### 3.9. LC-MS/MS Analysis of PIT

In this experiment, ESI-MS analysis used negative ion mode and identified the chemical constituents by comparing the profiles with authentic standards using the Multiple Reaction Monitoring (MRM) modes. Two pairs of MRM transitions were selected at m/z 353.1 → 191.0 and 515 → 353.  Table 1 displayed that 4-CQ, 5-CQ, 3,4-CQ, 3,5-CQ, and 4,5-CQ were detected in the PIT (Table 1). Moreover, the results showed a concentration of 3,5-CQ, which was the highest peak of the chromatogram at 169.93 *μ*g/ml of 1,500 *μ*g/ml PIT (data not showed).

## 4. Discussion

Free radicals encompassing the ROS and RNS are derived from both endogenous sources (mitochondria, peroxisomes, endoplasmic reticulum, phagocytic cells, etc.) and exogenous sources (pollution, alcohol, tobacco smoke, heavy metals, transition metals, industrial solvents, pesticides, certain drugs like halothane, paracetamol, and radiation) [31]. The imbalance between free radical and antioxidant systems can cause extensive damage to tissues and biomolecules [32], leading to various diseases especially degenerative diseases of aging such as cancer, immune-system decline, brain dysfunction, and cardiovascular [33]. Antioxidants derived from the diet assist physiological protective mechanisms in preventing damage from ROS or RNS. Dietary antioxidants can be derived from either supplements, fruit, vegetables, or herbal beverages, including teas. These are popular with consumers and are widely used to prevent the diseases generated by free radicals [34, 35].

PIT has been used for health-promoting tea, but its antioxidative properties have not been fully explored. In this study, the antioxidative properties of PIT were compared with a well-known and commercially available green tea. The properties were investigated using well-established techniques, including DPPH and ABTS. The properties of the tea to scavenge peroxynitrite, RNS, and hypochlorous acid and prevent the oxidation of isolated human LDL were also undertaken.

DPPH radical scavenging assay has been widely used in the determination of the antioxidant activity of natural antioxidants from plant sources [28, 36]. This assay determines the reduction of DPPH radical by measuring the color changing from the violet color of DPPH radical to yellow of the nonradical DPPH derivative at 515 nm. Several studies indicated that CST scavenged the DPPH radical [37, 38]. Interestingly, PIT showed significantly stronger antioxidant activity in this assay than CST at all concentrations. These results are in substantial agreement with Srisook et al. [39] that hot water extract of *P. indica* leaves shows the DPPH radical scavenging activity (EC_50_ value = 23.8 ± 1.0 *μ*g/ml).

ABTS^+^ radical cation decolorization assay was used to measure the antioxidant capacity of PIT compared to CST. These results provide evidence that CST could significantly reduce ABTS^+^ radical better than PIT. This higher action of CST may come from various classes of polyphenols in CST, which act as a potent antioxidant for the ABTS^+^ radical [40, 41].

Hypochlorous acid is a weak acid that could inactivate the antioxidant enzyme catalase by breaking down the heme prosthetic group [42]. Our results demonstrated that PIT was a higher potent hypochlorous acid scavenger than CST, which has been previously reported its hypochlorous acid scavenging capacity [43, 44].

Nitric oxide has an important role in several physiological processes like neural signal transmission, immune response, control vasodilation, and control of blood pressure [45, 46]. Nevertheless, the elevation of the nitric oxide causes inflammation and sustained levels of nitric oxide results in tissue toxicity and several pathological, including in vascular disease [47]. The present study examined the nitric oxide scavenging effect of PIT. Tsai et al. reported that CST had IC_50_ values of nitric oxide scavenging less than 500 *μ*g/ml and was proven to be a good nitric oxide suppressor [48]. Interestingly, our findings provide evidence that PIT displays significantly higher nitric oxide scavenging activity than CST.

Peroxynitrite, which is one of the nitrogen-containing species, is indicated as RNS. Excess peroxynitrite represents a crucial pathogenic mechanism in conditions, such as stroke, myocardial infarction, chronic heart failure, diabetes, circulatory shock, chronic inflammatory diseases, cancer, and neurodegenerative disorders [49]. PIT at a concentration of 75 *μ*g/ml had the capacity to fully inhibit the ability of peroxynitrite to bleach the color of Evan blue dye. Noticeably, PIT demonstrated significantly better peroxynitrite scavenging activity than the green tea preparation at the same concentration of 75 *μ*g/ml. Chung et al. reported that catechins, a galloyl group containing in green tea, inhibited peroxynitrite formation by both SIN-1 and scavenged peroxynitrite itself [50]. These findings lead us to believe that the effect of PIT on DPPH, ABTS, hypochlorous acid, nitric oxide, and peroxynitrite scavenging is better than CST.

LDL lipid oxidation is considered to be essential in the pathogenesis of atherosclerotic vascular diseases [51]. Several lines of evidence suggest that the important mechanisms of LDL lipid oxidation occur by ROS, RNS, and Cu^2+^ [52]. The natural compounds with anti-LDL oxidation activity could have some beneficial effects in the prevention of the disease [53, 54]. The investigation of anti-LDL oxidation activity *in vivo* can be measured *in vitro* by using whole plasma/serum [55]. In this study, we measured the LDL oxidation in AAPH, Cu^2+^, and SIN-1 challenged isolated human LDL using the TBARS assay and presented the results by MDA equivalence. AAPH is a ROS generator that can initiate lipid peroxidation and protein oxidation in isolated LDL particles. One measure of antioxidative protection in LDL is the lag-time. The inclusion of both PIT and CST extended the lag time compared to the control. From the results, the antioxidative properties of CST observed toward AAPH activity are consistent with those of Liu et al. reported that the polyphenolic components derived from green tea leaves are effective antioxidants against AAPH-initiated photosensitized LDL oxidation [56]. Noticeably, our studies found that PIT demonstrated better AAPH scavenging activity than green tea from 150 min onward. Besides, the Cu^2+^ scavenging activity effect of both PIT and CST was also investigated. The results expressed that PIT could favorably inhibit Cu^2+^ induced LDL oxidation compared to CST. It was reported by Yokozawa et al. that green tea extract markedly delayed Cu^2+^ induced LDL oxidation with a dose-dependent pattern [57]. Green tea contains a rich array of polyphenols, and these may chelate the copper ions from solution, the same may be true for PIT. Moreover, SIN-1, which is an RNS generator, was also used to investigate LDL oxidation. The RNS, which is generated by SIN-1, was scavenged by both teas. This result is consistent with the Evan blue dye study presented in this study. These results suggest that PIT may have a role in preventing the initial stages of atherogenic events by inhibiting ROS, RNS, and Cu^2+^ induce lipid peroxidation.

The PIT has been investigated as the main chemical constituent by using LC-MS/MS technique. The result expressed that caffeoylquinic acid derivatives were the main chemical compositions of PIT. This result is in correspondence with the study from Kongkiatpaiboon et al. [23], which reported that PIT contained six caffeoylquinic acid derivatives. Interestingly, caffeoylquinic acid derivatives were reported that they had potent antioxidant properties [58]. Caffeoylquinic acid derivatives from *Dipsacus asper* Wall (Dipsacaceae) showed antioxidant activity against free radical and Cu^2+^-mediated LDL oxidation. They may have an essential role in preventing the development and progression of atherosclerotic disease [58]. Furthermore, these derivatives also showed high DPPH-radical and peroxynitrite scavenging activity [59, 60]. These findings provide evidence that the antioxidative nature, which is free radical scavenging activities and anti-LDL oxidation, of PIT, is better than or approximately equal CST. These effects may be acted by caffeoylquinic acid derivatives rather than polyphenolic catechins found in CST [12].

## 5. Conclusion

The results of the present work show free radical scavenging activities and anti-LDL oxidation effects of PIT. The PIT has the potential to be developed as a health supplement product to provide antioxidants for atherosclerosis or other diseases associated with oxidative stress prevention. Further studies should focus on *in vivo* investigation, including pharmacokinetics, pharmacodynamic, efficacious, and safe dose in humans.

## Figures and Tables

**Table 1:** The main chemical constituent of *P. indica* tea. was analyzed by Liquid Chromatography-Mass Spectrometer/Mass Spectrometer (LC-MS/MS).

Main chemical constituent	Detection
4-O-caffeoylquinic acid (4-CQ)	+
5-O-caffeoylquinic acid (5-CQ)	+
3,4-O-dicaffeoylquinic acid (3,4-CQ)	+
3,5-O-dicaffeoylquinic acid (3,5-CQ)	+
4,5-O-dicaffeoylquinic acid (4,5-CQ)	+

## Data Availability

The data used and analyzed in this study are available from the corresponding author on reasonable request.
